# Transcriptomics and Metabolomics Combined to Analyze the Response Mechanism of Silkworm Eggs to High-Temperature Stress

**DOI:** 10.3390/insects16080862

**Published:** 2025-08-19

**Authors:** Yang Xiao, Qingrong Li, Zhenbo Sun, Bing Fu, Qiong Yang, Mangui Jiang, Weilong Zhang, Xuhua Huang, Dongxu Xing

**Affiliations:** 1 Sericultural & Agri-Food Research Institute, Guangdong Academy of Agricultural Sciences/Key Laboratory of Functional Foods, Ministry of Agriculture and Rural Affairs/Guangdong Key Laboratory of Agricultural Products Processing, Guangzhou 510610, China; xiaoyang@gdaas.cn (Y.X.); liqingrong@gdaas.cn (Q.L.); sunzhenbogd@163.com (Z.S.); 15869704640@163.com (B.F.); yangqiong@gdaas.cn (Q.Y.); zhangweilong@gdaas.cn (W.Z.); 2 Sericulture Technology Promotion Station of Guangxi/Guangxi Academy of Sericulture Sciences, Nanning 530007, China; jiangmg888@sina.com (M.J.); hgjsoil@163.com (X.H.)

**Keywords:** transcriptomics, metabonomics, silkworm egg, instant acid-impregnated eggs, high temperature stress

## Abstract

Conventional immediate and high-temperature immediate acid treatment are crucial techniques for breaking the diapause state of silkworm eggs, but their molecular mechanisms remain unclear. This study prepared diapause eggs (CK), conventional immediate acid-treated eggs (46 °C, 5 min, and CG), and high-temperature immediate acid-treated eggs (47.5 °C, 7 min, and GW) and analyzed the transcriptome and metabolome to screen for key expressed genes and key metabolites. Transcriptome results showed that 688, 823, and 222 differentially expressed genes (DEGs) were obtained from CK vs. CG, CK vs. GW, and CG vs. GW, respectively, and 12 DEGs significantly upregulated in all three comparisons (CK vs. CG, CK vs. GW, and CG vs. GW), including *glycine-N-methyltransferase*, *choline dehydrogenase*, *Hsp68*, and *Hsp70*. The LC-MS analysis results showed that 854, 711, and 506 differential metabolites (DMs) were obtained from CK vs. CG, CK vs. GW, and CG vs. GW, respectively. A total of seven DMs upregulated in all three comparisons and with |log_2_Fold Change| ≥ 0.5 in CG vs. GW, including tyrosine-isoleucine-histidine, phenylalanyl-tyrosine, tyrosine-phenylalanine-glutamate-lysine, and histidylleucine, as well as 12 downregulated DMs, were identified. Additionally, it was found that γ-linolenic acid and triglycerides were upregulated in CG vs. GW. The conjoint analysis results revealed that four small peptides, including tyrosine-isoleucine-histidine, phenylalanyl-tyrosine, tyrosine-phenylalanine-glutamate-lysine, and histidylleucine, exhibited a highly significant positive correlation with *Hsp70* family genes such as *Hsp68* and *Hsp70*. This suggests that these small peptides, along with γ-linolenic acid and triglycerides, may play a crucial role in the resistance of silkworm eggs to high-temperature stress and the associated oxidative stress.

## Simple Summary

Silkworm eggs will enter a state of diapause (such as deep sleep), and the hydrochloric acid hatching method can prevent them from entering this state. Currently, the conventional treatment method used in sericultural production is to immerse silkworm eggs in a 1.075 g/mL hydrochloric acid solution at 46 °C for 5 min. We found that increasing the temperature and duration (47.5 °C for 7 min) not only prevents diapause but also inactivates pathogens in silkworm eggs. However, high temperatures can also lead to a decrease in the hatching rate of silkworms. Therefore, understanding the molecular mechanisms underlying the response of silkworm embryos to high-temperature stress is crucial for optimizing this dual-use treatment. By comparing diapause eggs with those treated with the two methods, we found 12 genes that became more active, including some that produce proteins helping cells handle stress. We also found small molecules like certain protein fragments, a type of acid, and fats, which seemed linked to these stress-handling genes. These findings suggest that these genes and molecules help silkworm eggs survive high temperatures and related damage. This can improve sericulture and help us understand how other insects cope with high-temperature challenges such as rising temperatures.

## 1. Introduction

Insects are ectotherms, and their growth, development, physiological status, behavioral activities, and ecological adaptability are closely related to environmental temperatures [1,2]. Numerous studies have shown that high-temperature stress has negative impacts on insect survival rate, fecundity, the hatching rate of offspring, pupation rate, eclosion rate, etc. [3,4,5,6,7,8], which are detrimental to their growth, development, and reproduction. Under the background of global warming, the frequency and intensity of extreme high temperatures have increased significantly, meaning that insects will face more and more high-temperature stress [9,10,11]. Therefore, understanding the response mechanism of insects to high-temperature stress is of great significance for evaluating the potential of biocontrol insects, predicting the occurrence of agricultural pest insects, and ensuring the breeding of economic insects.

The silkworm (*Bombyx mori*) is a typical insect that undergoes egg diapause, a state in which embryonic development is arrested, serving as a crucial adaptive mechanism to cope with adverse environmental conditions. In sericultural production, the hydrochloric acid hatching method is the most commonly used artificial technique for terminating silkworm egg diapause. Originating in Italy and France in the 19th century, this method was established as a practical artificial hatching technology for silkworm eggs in Japan in 1914. Specifically, it involves soaking silkworm eggs in a 1.075 g/mL hydrochloric acid solution at 46 °C for 5 min, which effectively breaks their diapause [12]. In addition to its role in the termination of diapause, high-temperature treatment (>47 °C) can also control the vertical transmission of *Nosema bombycis* in silkworms. It has been shown that acid treatment at 47.5 °C for 7 min can significantly inhibit the proliferation of *N. bombycis* within eggs and reduce the risk of vertical transmission [13]. However, excessive high temperatures can also lead to a decrease in the hatching rate of silkworms. Therefore, understanding the molecular mechanisms underlying the response of silkworm embryos to high temperature-stress is crucial for optimizing this dual-use treatment. Moreover, the silkworm is a lepidopteran model insect that has completed the genome project [14], making it an excellent biological material for studying the response mechanism of insect eggs to high-temperature stress.

Within a certain temperature range, insects can tolerate high-temperature stress through a series of physiological and biochemical reactions to ensure a normal life history. High-temperature stress often induces the production of a large number of reactive oxygen species (ROS) in the body, leading to a sharp increase in free radical content and causing oxidative damage [15,16]. To reduce the oxidative level, insects enhance the activity of protective enzyme systems such as superoxide dismutase, catalase, peroxidase, and glutathione-S-transferase to scavenge excessive ROS [17,18,19,20]. When insects are subjected to high-temperature stress, cells in the body can also induce the synthesis of heat shock proteins (HSPs), which act as molecular chaperones to protect protein integrity and cellular homeostasis [21]. According to molecular weight and homology, *Hsps* are divided into *sHsps*, *Hsp60*, *Hsp70*, and *Hsp90* [22,23]. Studies have shown that the heat tolerance of insects is positively proportional to the expression level of *Hsps* [24,25,26]. After high-temperature treatment, the transcriptional levels of *AiHsp19.3*, *HcHsp20.1*, and *HcHsp21.5* in *Agrotis ipsilon* and *Hyphantria cunea* increased significantly [27,28]. *Leptinotarsa decemlineata* and *Sitodiplosis mosellana* respond to high-temperature stimulation by promoting the expression of *Hsp60* [29,30]. Under high-temperature stress, the expression levels of *Hsp70* in *Callosobruchus chinensis* and *Arma chinensis* were significantly upregulated compared with the control group [31,32]. The expression of *Hsp90* in *Antheraea assamensis* and *Tyrophagus putrescentiae* increased significantly after high-temperature stress [33,34], indicating that four types of HSPs, including sHSPs, HSP60, HSP70, and HSP90, all play roles in insect resistance to high-temperature stress. In addition to HSPs, insects also promote the expression of stress-resistant substances such as trehalose, glycogen, sorbitol, triglyceride, and linolenic acid to improve their heat resistance [35,36,37,38,39,40].

According to previous studies, within a certain temperature range, high-temperature stress on insect eggs often accelerates their development rate and shortens the developmental duration. At 32 °C, the time required for eggs of *Hieroglyphica shibinensis* to develop into adults was 14.17 d shorter than that at 24 °C, and the average developmental duration of eggs was the shortest [41]. Within the range of 20–32 °C, the hatching speed of *Callosobruchus maculatus* eggs increased, and the hatching time gradually decreased with the rise in temperature [42]. The hatching time of eggs of Aethina tumida at 35 °C was 39 h shorter than that at 21 °C [43]. In addition, insects such as *Trichogramma dendrolimi*, *Aphytis japonicus*, *Archips podanus*, *Mesoneura rufonota*, and *Bactrocera dorsalis* also exhibit the above characteristics [44,45,46,47,48], indicating that high-temperature stress has a significant impact on insect eggs. However, the mechanism of insect eggs responding to high-temperature stress, including silkworm eggs, has been rarely reported and needs in-depth study.

Previous studies on immediate acid soaking with the heating of silkworm eggs mainly focused on the optimization of methods and the mechanisms related to diapause termination, but the response mechanism of silkworm eggs to high temperatures remains unclear. Using diapaused eggs as a control, RNA-seq high-throughput sequencing and liquid chromatography–mass spectrometry (LC-MS) were used to compare and analyze the transcriptomic and metabolomic differences in silkworm eggs under conventional immediate acid soaking (46 °C, 5 min) and high-temperature immediate acid soaking (47.5 °C, 7 min). Through joint analysis, key genes and key metabolites of silkworm eggs responding to high-temperature stimulation were mined, providing a theoretical reference for the response mechanism of insect eggs to high-temperature stress.

## 2. Materials and Methods

### 2.1. Test Materials

The silkworm variety used in the test was the orthogonal strain of the bivoltine tetrahybrid “Liangguang No. 2” (9·Fu × 7·Xiang), supplied by Yangshan Branch of Guangdong Siyuan Group Co., Ltd. (Yangshan, China). At 20 h after egg laying, silkworm eggs were subjected to conventional immediate acid soaking (1.075 g/mL HCL, treatment at 46 °C for 5 min, and CG) and high-temperature immediate acid soaking (1.075 g/mL HCL, treatment at 47.5 °C for 7 min, and GW), with diapaused eggs as the control group (CK). Each group included 12 egg circles, which were eluted into loose eggs and then incubated at 27 ± 1 °C with a relative humidity of 80–85%. At 32 h after egg laying, two pieces of 0.9 g silkworm eggs were taken from each group, quickly frozen in liquid nitrogen, and stored in a −80 °C refrigerator. Subsequent experiments were conducted by Wuhan Besino Biotechnology Co., Ltd. (Wuhan, China).

### 2.2. Transcriptome Sequencing and Analysis

#### 2.2.1. RNA Extraction and Detection

Silkworm egg samples were divided into 3 replicates for each group, and RNA was extracted using a Trizol kit (Thermo Fisher Scientific, Waltham, MA, USA). The integrity, purity, and concentration of RNA were detected by agarose gel electrophoresis, Qsep400 bioanalyzer (Bioptic, Guangding Biotechnology (Jiangsu) Co., Ltd., Changzhou, China), NanoPhotometer spectrophotometer (Implen GmbH, Munich, Germany), and Qubit 4.0 fluorometer/MD microplate reader (Thermo Fisher Scientific, Waltham, MA, USA).

#### 2.2.2. Transcriptome Sequencing

Oligo (dT) magnetic beads were used to enrich the extracted sample RNA to obtain mRNA with polyA tails. Fragmentation buffer was added to break the RNA into short fragments. Using these short RNA fragments as templates, first-strand cDNA was synthesized with random hexamer primers. Then, buffer, dNTPs (dTTP, dATP, dGTP, and dCTP), and DNA polymerase I were added to synthesize the second strand cDNA. The double-strand cDNA was purified using DNA purification magnetic beads. The purified double-strand cDNA was subjected to end repair, A-tailing, and the ligation of sequencing adapters, followed by fragment size selection using DNA purification magnetic beads, and finally, PCR enrichment to generate the final cDNA library. After passing library inspection, sequencing was performed on an Illumina platform to obtain paired-end reads.

#### 2.2.3. Data Quality Control and Sequence Alignment

Raw sequencing data were filtered using Fastp to remove adapter-containing reads, yielding clean reads. The silkworm genome database (https://silkdb.bioinfotoolkits.net/base/download/-1 (accessed on 3 August 2023)) was used as the reference genome, and HISAT2 was used to align the clean reads to the reference genome.

#### 2.2.4. Gene Expression Level Quantification and Differential Analysis

FPKM (Fragments Per Kilobase of transcript per Million fragments mapped) was used as an indicator to measure the expression level of transcripts or genes. DESeq2 was applied for a differential expression analysis between sample groups. Differentially expressed genes (DEGs) were screened under the criteria of |log_2_Fold Change| ≥ 1 and FDR < 0.05. Based on GO terms, DEGs were classified through GO annotation. Additionally, after annotating DEGs to the KEGG database, a KEGG enrichment analysis was conducted using the hypergeometric test with KEGG pathways as the units.

#### 2.2.5. RT-qPCR Validation

According to the study by Guo et al. [49], *GAPDH* was used as the internal reference gene. Eight DEGs were selected for RT-qPCR relative expression analysis. Primers were designed using Premier 5.0 software (Table S1), and the 2^−△△Ct^ method was employed to calculate gene expression levels [50]. Total RNA of silkworm eggs was extracted using a Trizol kit (Thermo Fisher Scientific, Waltham, MA, USA). After detecting the concentration and quality, reverse transcription was performed. The reverse transcription kit PrimeScript™ RT reagent Kit with gDNA Eraser (Perfect Real Time) (Code No. RR047A) and the fluorescence quantitative kit SYBR^®^ Premix Ex Taq™ II (Tli RNaseH Plus) (Code No. RR820A) were both purchased from TaKaRa Biomedical Co., Ltd., Beijing, China. The fluorescence quantitative PCR utilized a 20 μL reaction system: 10 μL SYBR^®^ Premix Ex Taq II (Tli RNaseH Plus) (2×), 2 μL DNA template, 0.8 μL of each upstream and downstream primer, and 6.4 μL ddH_2_O. The reaction program was as follows: pre-denaturation at 95 °C for 30 s; 40 cycles of 95 °C for 5 s and 60 °C for 30 s.

### 2.3. Metabolomic Analysis

#### 2.3.1. Metabolome Sample Extraction

Samples were retrieved from the −80 °C refrigerator with each divided into 3 replicates and thawed on ice. Samples were homogenized under liquid nitrogen, and 20 mg of the homogenized sample was weighed into a labeled centrifuge tube. A total of 400 μL of 70% methanol–water solution containing an internal standard was added, followed by oscillation at 1500 r/min for 5 min and incubation on ice for 15 min. The mixture was centrifuged at 12,000 r/min for 10 min at 4 °C, and 300 μL of the supernatant was transferred to another labeled centrifuge tube, then stored at −20 °C for 30 min. After re-centrifugation at 12,000 r/min for 3 min at 4 °C, 200 μL of the supernatant was transferred to the insert of a corresponding autosampler vial for instrumental analysis.

#### 2.3.2. LC-MS Analysis

LC-MS analysis was performed based on the Maiwei Metabolomics Platform. Liquid Chromatography Conditions: Waters ACQUITY Premier HSS T3 Column (1.8 µm, 2.1 mm × 100 mm, Milford, CT, USA); mobile phase A was ultrapure water (with 0.1% formic acid), mobile phase B was acetonitrile (with 0.1% formic acid); elution gradient: 5% B at 0.0 min, 20% B at 2.0 min, 60% B at 5.0 min, 99% B from 6.0 to 7.5 min, and 5% B from 7.6 to 10.0 min; and flow rate was 0.4 mL/min, column temperature was 40 °C, and injection volume was 4 μL. Mass Spectrometry Conditions: Electrospray ionization (ESI) in positive and negative ion modes was used for detection; ion source temperature was 550 °C (ESI+) and 450 °C (ESI−).

#### 2.3.3. Metabolite Identification and Quantitative Analysis

Raw data were converted to the mzXML format using ProteoWizard software (v3.0.8789), and peak extraction, alignment, and retention time corrections were carried out using the XCMS program (v3.12.0). The “SVR” method was used to correct peak areas, and peaks with a missing rate > 50% in each group were excluded. After correction and filtering, metabolite identification information was achieved by searching self-built databases, integrating public libraries, AI prediction libraries, and the metDNA method. Differential metabolites (DMs) were selected by combining the variable importance in the projection (VIP) value from the OPLS-DA model with the *p* value of the *t*-test, with the criteria of *p* < 0.05 and VIP > 1.

#### 2.3.4. Integrated Analysis of Transcriptome and Metabolome Integrated Transcriptome and Metabolome Analysis

Based on the DMs analysis results of this study, combined with the differential gene analysis results, DEGs and DMs from the same comparison group were jointly mapped to KEGG pathway maps. Correlation analysis was performed using the quantitative values of genes and metabolites in all samples, and the Pearson correlation coefficient between genes and metabolites was calculated using the cor function in R. The screening threshold was set as an absolute correlation coefficient > 0.8 and *p*-value < 0.05.

## 3. Results and Analysis

### 3.1. Transcriptomic Analysis of Silkworm Embryo Response to High-Temperature Stress

#### 3.1.1. Sequencing Data Quality Control

In this study, transcriptome sequencing was performed on CK, CG, and GW silkworm egg samples. The Q30 value of each sample was above 93.35%, and the GC content ranged from 41.85% to 43.74%. By matching with the silkworm genome database, the alignment efficiency of reads from each sample to the silkworm genome was between 87.04% and 89.19% (Table S2). The results show that the sequencing data have a good quality, meeting the requirements for subsequent analysis.

#### 3.1.2. Statistics of DEGs

**Figure 1:**
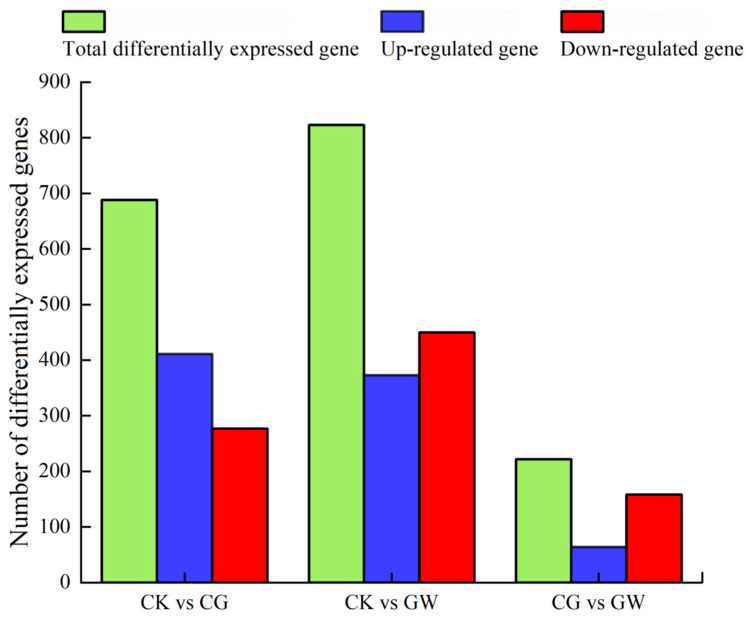
Statistics of DEGs among each group. CK, control group, and 28 °C; CG, conventional instant acid-impregnated, 46 °C, and 5 min; and GW, high-temperature instant acid-impregnated, 47.5 °C, and 7 min.

Based on the differential expression thresholds of |log2Fold Change| ≥ 1 and FDR < 0.05, DEGs among the three groups (CK, CG, and GW) were statistically analyzed (Figure 1). In CK vs. CG, a total of 688 DEGs were identified, including 411 significantly upregulated genes and 277 significantly downregulated genes. In CK vs. GW, a total of 823 DEGs were identified, including 373 significantly upregulated genes and 450 significantly downregulated genes. In CG vs. GW, a total of 222 DEGs were identified, including 64 significantly upregulated genes and 158 significantly downregulated genes.

#### 3.1.3. GO Functional Classification of DEGs

**Figure 2:**
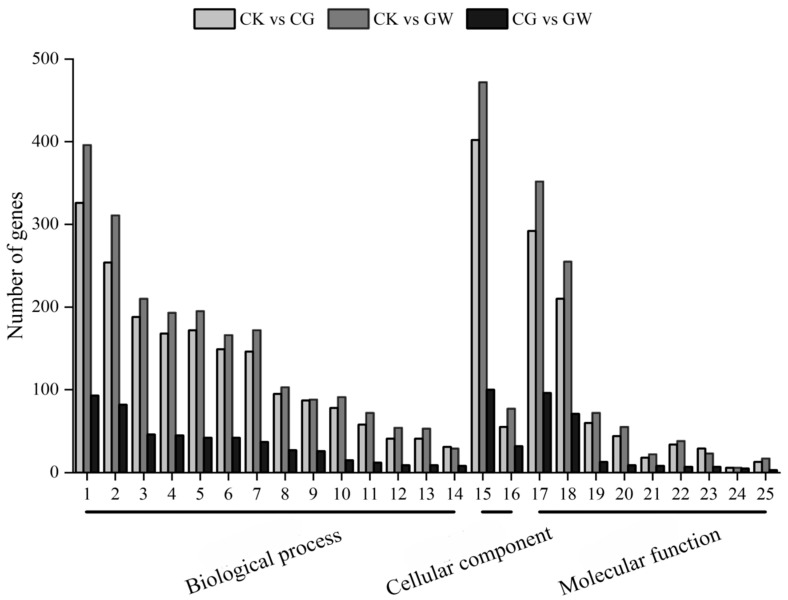
GO functional annotation of DEGs in each group. CK, control group, and 28 °C; CG, conventional instant acid-impregnated, 46 °C, and 5 min; and GW, high-temperature instant acid-impregnated, 47.5 °C, 7 min.

Based on the GO database, a functional annotation and cluster analysis were performed on DEGs. DEGs in the three groups (CK vs. CG, CK vs. GW, and CG vs. GW) were mostly distributed in the same GO ontology terms. Among them, GO ontology terms such as “cellular process” and “metabolic process” in the biological process category, “cellular anatomical integrity” in the cellular component category, and “binding” and “catalytic activity” in the molecular function category were significantly enriched with a large number of DEGs (Figure 2). The results indicate that DEGs in CK vs. CG, CK vs. GW, and CG vs. GW show similar GO functional distributions.

#### 3.1.4. KEGG Pathway Enrichment Analysis of DEGs

**Figure 3:**
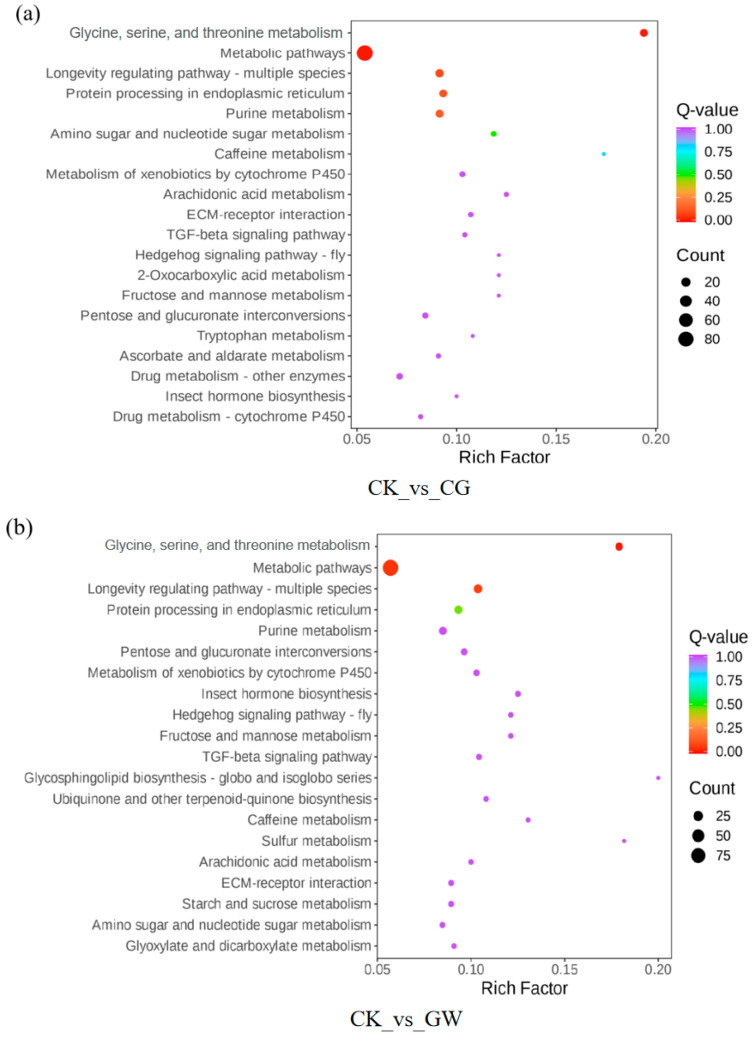

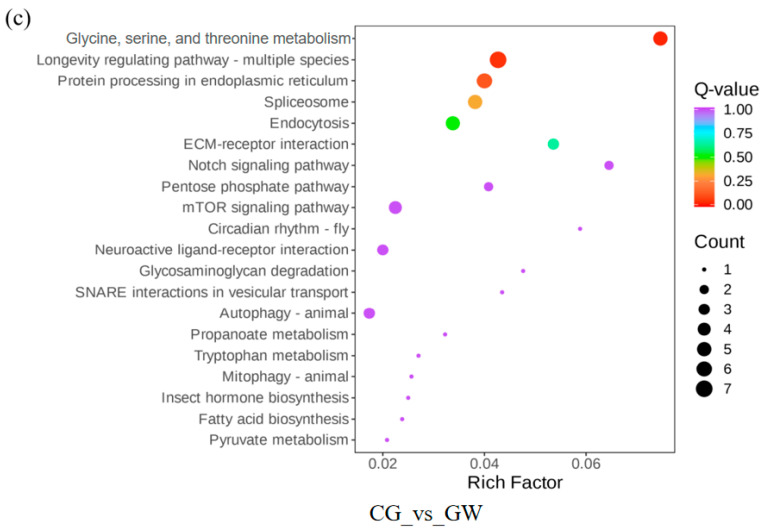
KEGG signaling pathway enrichment analysis of DEGs in each group. (**a**) CK vs. CG; (**b**) CK vs. GW; and (**c**) CG vs. GW. CK, control group, and 28 °C; CG, conventional instant acid-impregnated, 46 °C, and 5 min; and GW, high-temperature instant acid-impregnated, 47.5 °C, and 7 min.

A KEGG pathway enrichment analysis was performed on DEGs based on the KEGG database. The number of DEGs enriched at different hierarchical levels of each KEGG pathway was counted to determine the main metabolic pathways involved. The results showed that DEGs in both CK vs. CG (Figure 3a) and CK vs. GW (Figure 3b) were significantly enriched in the “Glycine, serine, and threonine metabolism” and “Metabolic pathways” pathways. In contrast, DEGs in CG vs. GW were significantly enriched in the “Glycine, serine, and threonine metabolism” and “Longevity regulating pathway—multiple species” pathways (Figure 3c). The “Glycine, serine, and threonine metabolism” pathway, being the common significantly enriched pathway in CK vs. CG, CK vs. GW, and CG vs. GW, may be closely related to the high-temperature stress response of silkworm eggs.

#### 3.1.5. Screening and Identification of DEGs

Regarding the “Glycine, serine, and threonine metabolism” pathway, screening revealed that the *glycine-N-methyltransferase* gene and *choline dehydrogenase* gene were commonly enriched in all three gene comparisons (CK vs. CG, CK vs. GW, and CG vs. GW), both of which were upregulated. Additionally, analysis of *Hsp* genes identified a total of 10 DEGs, including *Hsp68 isoform X1*, *Hsp68*, *Hsp19.9*, *Hsp20.8*, *Hsp20.4*, *Hsp70 A1*, *Hsp70*, and *Hsp23.7* precursor, all of which were upregulated in all three comparisons (Table 1). Among them, five *Hsp70* family genes (two *Hsp68*, two *Hsp70*, and *Hsp70 A1*) were commonly and significantly upregulated.

#### 3.1.6. RT-qPCR Validation of Transcriptome Data

**Figure 4:**
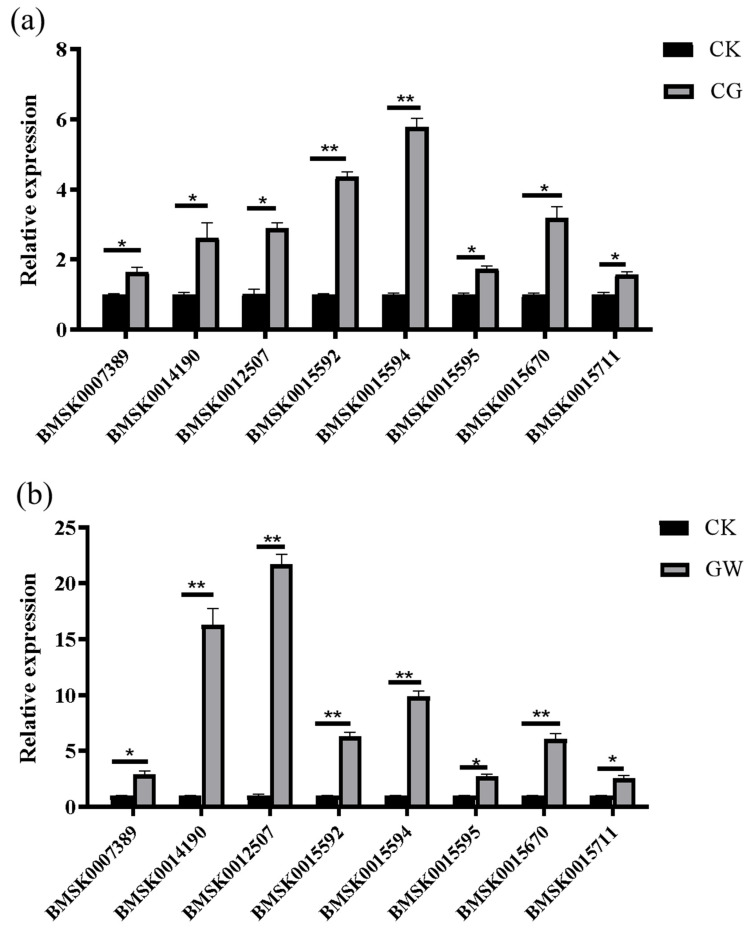

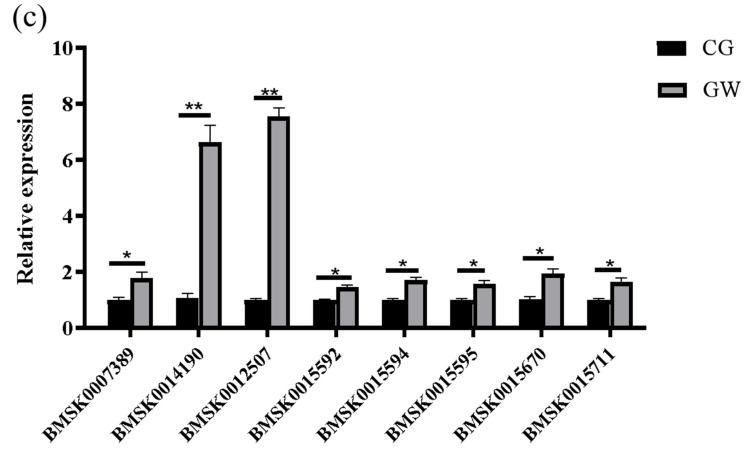
Verification of DEGs in each group. (**a**) CK vs. CG; (**b**) CK vs. GW; and (**c**) CG vs. GW. CK, control group, and 28 °C; CG, conventional instant acid-impregnated, 46 °C, and 5 min; and GW, high-temperature instant acid-impregnated, 47.5 °C, and 7 min. The figure column with * indicates *p* < 0.05, and the column with ** indicates *p* < 0.01.

RT-qPCR results showed that the expression trends of *glycine-N-methyltransferase*, *choline dehydrogenase*, *Hsp68*, *Hsp19.9*, *Hsp20.8*, *Hsp20.4*, *Hsp70*, and *Hsp23.7* precursors were basically consistent with the transcriptome data, indicating the high reliability of the transcriptome results (Figure 4).

### 3.2. Metabolomic Analysis of Silkworm Embryo Response to High-Temperature Stress

#### 3.2.1. Metabolic Profile Analysis

**Figure 5:**
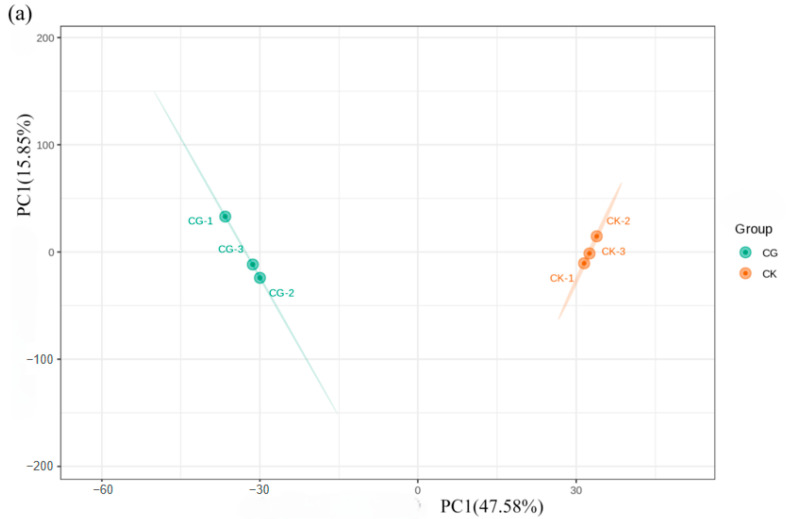

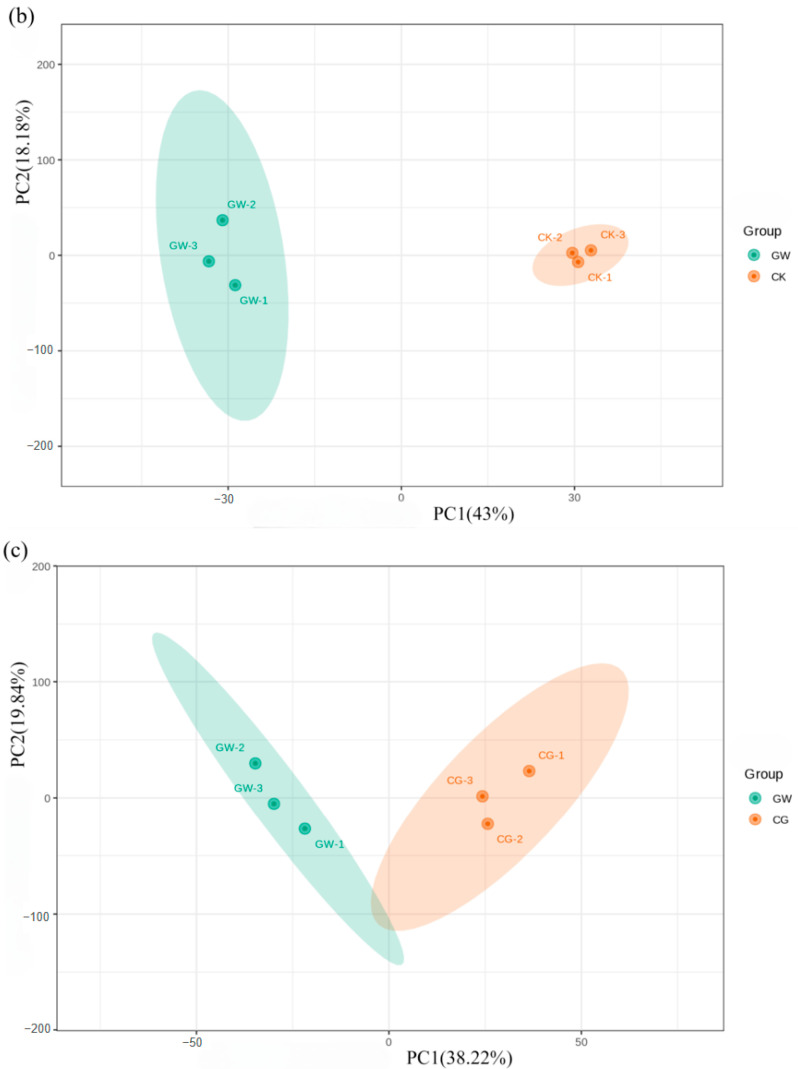
Principal component analysis in each group. (**a**) CK vs. CG; (**b**) CK vs. GW; and (**c**) CG vs. GW. CK, control group, and 28 °C; CG, conventional instant acid-impregnated, 46 °C, and 5 min; and GW, high-temperature instant acid-impregnated, 47.5 °C, and 7 min.

The principal component analysis (PCA) model showed that for CK and CG, the first principal component (PC1) accounted for 47.58%, and the second principal component (PC2) accounted for 15.85% (Figure 5a). For CK and GW, PC1 accounted for 43% and PC2 accounted for 18.18% (Figure 5b). For CG and GW, PC1 accounted for 38.22% and PC2 accounted for 19.84% (Figure 5c). Three replicate samples in each test group clustered together, indicating a good repeatability, while samples from different groups were discrete, suggesting differences in their metabolomes.

#### 3.2.2. Statistics of DMs and KEGG Pathway Enrichment Analysis

**Figure 6:**
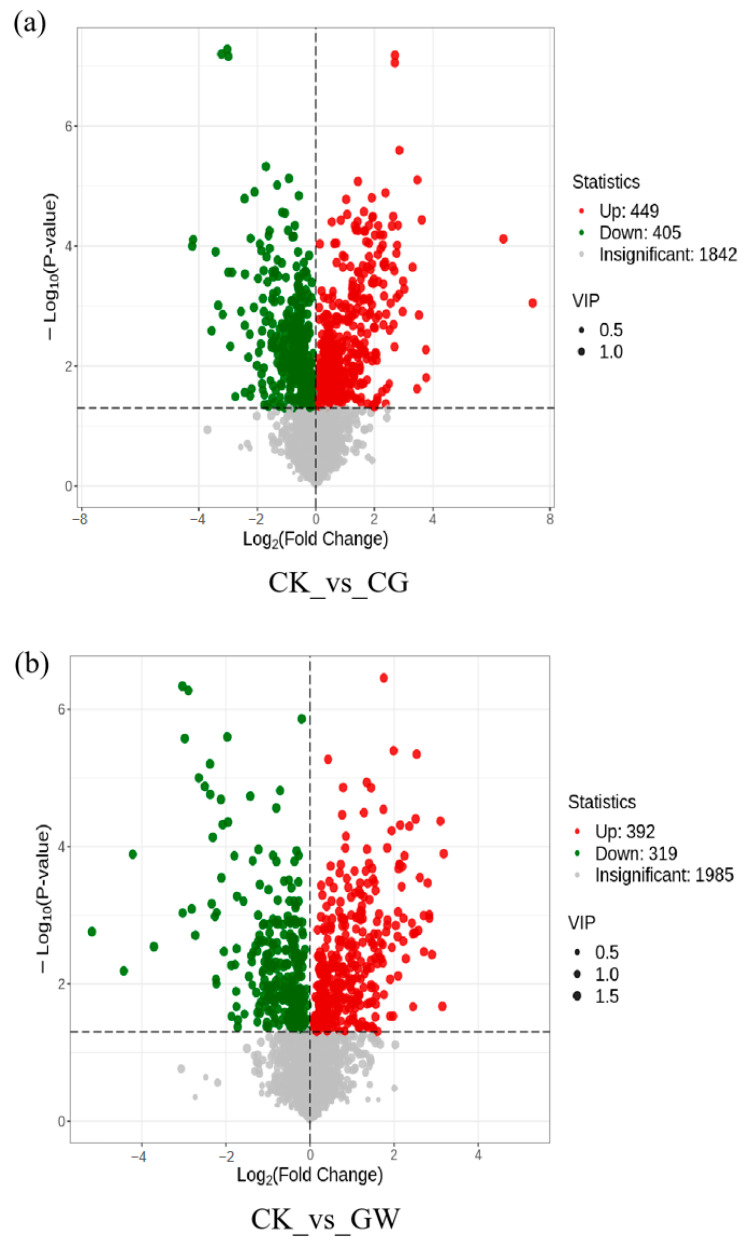

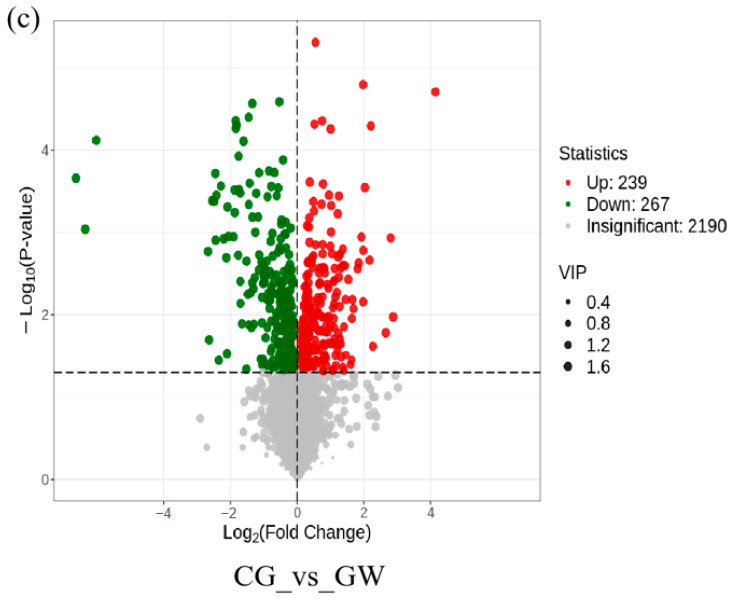
Volcano plot of DMs in each group. (**a**) CK vs. CG; (**b**) CK vs. GW; and (**c**) CG vs. GW. CK, control group, and 28 °C; CG, conventional instant acid-impregnated, 46 °C, and 5 min; and GW, high-temperature instant acid-impregnated, 47.5 °C, and 7 min.

**Figure 7:**
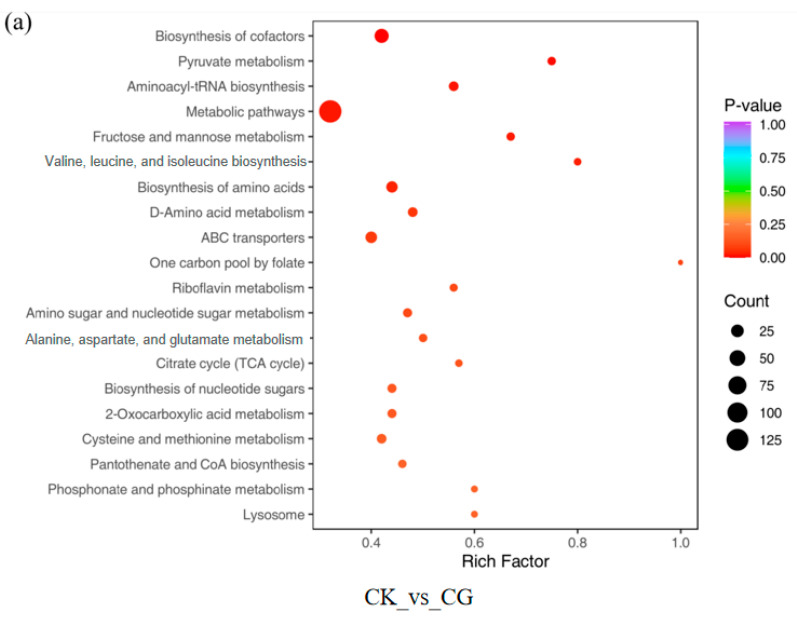

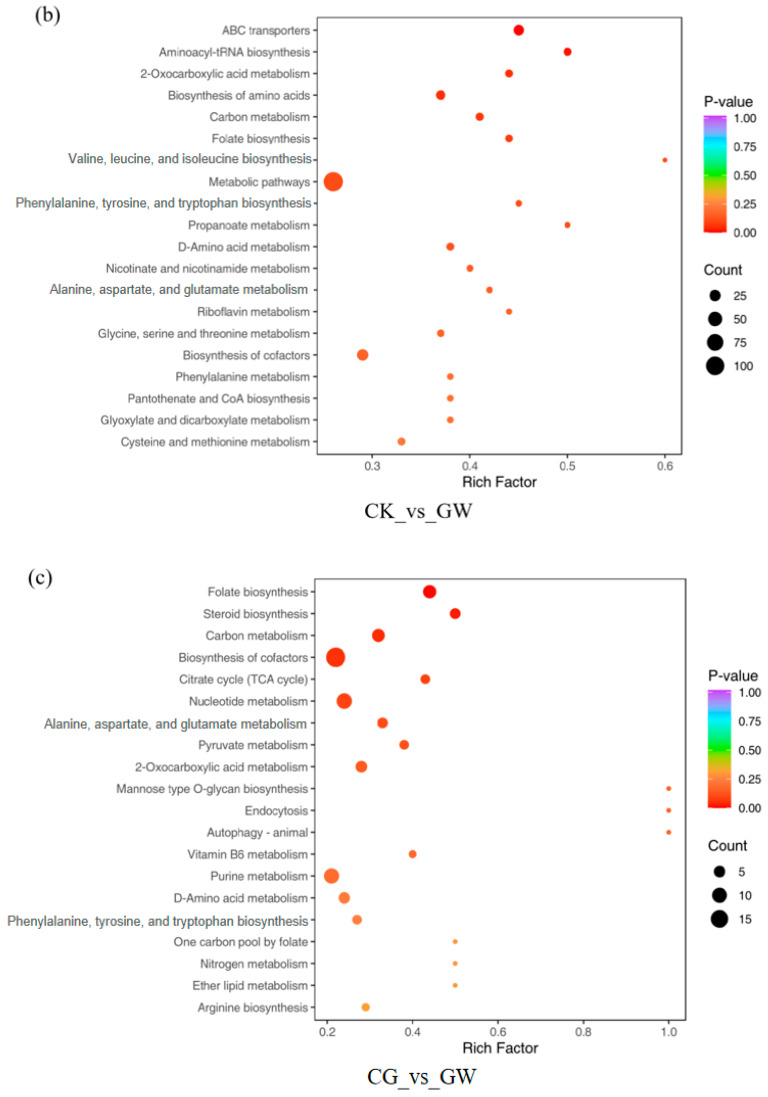
KEGG signal pathway enrichment analysis of DMs in each group. (**a**) CK vs. CG; (**b**) CK vs. GW; and (**c**) CG vs. GW. CK, control group, and 28 °C; CG, conventional instant acid-impregnated, 46 °C, and 5 min; and GW, high-temperature instant acid-impregnated, 47.5 °C, and 7 min.

Using the OPLS-DA model and *t*-test, with screening criteria of *p* < 0.05 and VIP > 1, DMs in CK vs. CG, CK vs. GW, and CG vs. GW were obtained. CK vs. CG had 854 DMs, including 449 upregulated and 405 downregulated (Figure 6a), significantly enriched in seven KEGG pathways: “Biosynthesis of cofactors”, “Pyruvate metabolism”, “Aminoacyl-tRNA biosynthesis”, “Metabolic pathways”, “Fructose and mannose metabolism”, “Valine, leucine and isoleucine biosynthesis”, and “Biosynthesis of amino acids” (Figure 7a). CK vs. GW had 711 DMs, including 392 upregulated and 319 downregulated (Figure 6b), significantly enriched in three KEGG pathways: “ABC transporters”, “Aminoacyl-tRNA biosynthesis”, and “2-Oxocarboxylic acid metabolism” (Figure 7b). CG vs. GW had 506 DMs, including 239 upregulated and 267 downregulated (Figure 6c), significantly enriched in three KEGG pathways: “Folate biosynthesis”, “Steroid biosynthesis”, and “Carbon metabolism” (Figure 7c). The results showed that CK vs. CG and CK vs. GW were both significantly enriched in the “Aminoacyl-tRNA biosynthesis” pathway, but no common significantly enriched pathway was found among the three comparison groups. The results showed that CK vs. CG and CK vs. GW were both significantly enriched in the “Aminoacyl-tRNA biosynthesis” pathway, but no common significantly enriched pathway was found among the three comparison groups.

#### 3.2.3. Screening and Identification of DMs

Analysis of DMs in CK vs. CG, CK vs. GW, and CG vs. GW revealed 30 common upregulated metabolites and 26 common downregulated metabolites in all three comparisons. Using |log_2_Fold Change| ≥ 0.5 in CG vs. GW as the screening threshold, the identified common upregulated key metabolites included tyrosine-isoleucine-histidine, 2-hexadecylthio-1-ethylphosphorylcholine, N-(6-aminohexanoyl)-6-aminohexanoate, pantetheine, phenylalanyltyrosine, tyrosine-phenylalanine-glutamic acid-lysine, and histidylleucine. Common downregulated metabolites included docosahexaenoic acid-d5, carnitine C18:3-OH, methylephedrine, (9S,10S,12Z)-9,10-dihydroxy-12-octadecenoic acid, ethyl myristate, (9Z,12S,13R)-12,13-dihydroxy-9-octadecenoic acid, cetylpyridine, bisphenol A diglycidyl ether, glutamic acid-valine-tyrosine, cysteine protease-3 inhibitor Ac-DEVD-CHO, 9-ketoflutamide, and 3-sulfate resveratrol. Additionally, based on previous reports, stress-resistant substances related to heat stress, such as γ-linolenic acid and triglycerides, were found to be upregulated in CG vs. GW (Table 2).

### 3.3. Integrated Transcriptome and Metabolome Analysis

An integrated analysis of the screened DEGs and DMs was performed to explore their correlation in CG vs. GW. The results showed that four small peptides (tyrosine-isoleucine-histidine, phenylalanyltyrosine, tyrosine-phenylalanine-glutamic acid-lysine, and histidylleucine) had a correlation coefficient > 0.8 and *p* < 0.05 with *Hsp70* family genes (*Hsp68*, *Hsp70 A1*, and *Hsp70*), indicating a high positive correlation between small peptides and *Hsp70* family genes (Table 3).

## 4. Discussion

In this study, diapaused eggs (CK), conventional immediate acid-soaked eggs (CG), and high-temperature immediate acid-soaked eggs (GW) were used as research objects. Three comparison groups (CK vs. CG, CK vs. GW, and CG vs. GW) were designed, and the effects of high-temperature stress on gene expression and metabolic changes in silkworm eggs were revealed through an integrated transcriptome and metabolome analysis. Considering that DEGs or DMs in CK vs. CG and CK vs. GW might be caused by different developmental states, this study focused on the common DEGs and DMs among the three groups (CK vs. CG, CK vs. GW, and CG vs. GW). The mined genes and metabolites are closely related to high-temperature stress. Finally, a Pearson correlation coefficient analysis showed that four small peptides (tyrosine-isoleucine-histidine, phenylalanyltyrosine, tyrosine-phenylalanine-glutamic acid-lysine, and histidylleucine) had extremely significant positive correlations with *Hsp70* family genes (*Hsp68*, *Hsp70 A1*, and *Hsp70*), indicating that small peptides and *Hsp70* play important roles in the high-temperature stress response of silkworm eggs.

Hsp70 is the most sensitive type of heat shock protein to environmental stress and the most common Hsp in the high-temperature stress response of insects [51,52,53,54]. After the microinjection of dsRNA significantly reduced the expression of the *P-Hsp70* gene, the average lifespan of *Propylaea quatuordecimpunctata* under high temperatures (32–38 °C) was significantly shortened [55]. When the expression of *Aphis gossypii*’s *ApHsp70A1-1* and *ApHsp68* genes was inhibited, its sensitivity to high temperatures increased, and the adult survival rate at 29–35 °C was significantly lower than that of the control group [56]. After *Hsp70* was silenced by RNAi, the fecundity, hatching rate, and survival rate of the offspring of *Agasicles hygrophila* treated at 36 °C and 39 °C were significantly lower than those of the control group [57]. Similarly, this study found that five *Hsp70* family genes were significantly upregulated in all three groups (CK vs. CG, CK vs. GW, and CG vs. GW), further confirming that *Hsp70* and *Hsp68* are key genes for silkworm eggs to respond to high-temperature stress. Through the screening and identification of DMs and integrated transcriptome-metabolome analysis, four small peptides were found to have extremely significant positive correlations with *Hsp70* family genes.

Small peptides, composed of 2–20 amino acids, are rich in variety and diverse in function, capable of regulating biological processes such as cell proliferation, differentiation, and apoptosis [58]. And they also have strong antioxidant activity, which can alleviate the oxidative damage induced by high and low-temperature stress [59,60]. For insects, studies have shown that small peptides from *Periplaneta americana* can reduce the content of oxygen free radicals and nitric oxide free radicals, inhibiting H_2_O_2_-induced cellular oxidative stress damage and apoptosis [61,62]. The antioxidant activity of small peptides is related to their amino acid composition, and tyrosine, histidine, leucine, phenylalanine, lysine, glutamic acid, etc., all contribute to antioxidant activity [63,64,65]. The four small peptides identified in this study all contain the above amino acids, indicating that these small peptides play an important role in reducing the oxidative damage induced by high-temperature stress in silkworm eggs. In addition, *Hsps* protect proteins and polypeptides from denaturation during high-temperature stress [21,66]. The high positive correlation between small peptides and *Hsp70* family genes in the integrated analysis suggests that *Hsp70* plays an important protective role in small peptides. Therefore, this study suggests that in the face of high-temperature stress and induced oxidative stress, silkworm eggs can increase the expression levels of *Hsp70* and small peptides, providing support for life activities such as effective protein utilization and normal cell proliferation. However, specific functional verification requires further in-depth research.

Meanwhile, as stress-resistant substances related to heat stress [18,35], γ-linolenic acid and triglycerides, were upregulated in CG vs. GW, they were downregulated in CK vs. CG and CK vs. GW. It is speculated that during the metabolic dormancy of diapause eggs (CK), energy reserves are primarily in the form of glycogen, whereas after the termination of diapause, lipids need to be rapidly mobilized to provide energy for embryonic development. We believe that γ-linolenic acid and triglycerides still play a role in resisting high temperatures in silkworm eggs: On the one hand, γ-linolenic acid not only participates in the construction of biological membranes but also has stronger antioxidant activity in lipid environments; on the other hand, the increase in lipid content in silkworm eggs can effectively prevent water loss and reduce the death of silkworm eggs under high-temperature stress [35,67,68].

In addition to *Hsp70* family genes and small peptides, this study also screened two DEGs (*glycine-N-methyltransferase* gene and *choline dehydrogenase* gene) and 15 DMs. *Glycine-N-methyltransferase* is involved in glycine metabolism and also plays a significant role in liver fibrosis, steatosis, and neural differentiation [69]. *Choline dehydrogenase* catalyzes the formation of betaine from choline and acetic acid. Betaine helps maintain cellular water balance in high-temperature and high-salt environments, while activating the Nfr2 pathway to alleviate oxidative stress [70,71]. However, in the combined transcriptome–metabolome analysis, no significant changes were observed in the corresponding metabolites or key enzymes. The high-temperature resistance process in insects is very complex, and the mechanism of insect eggs responding to high-temperature stress has been rarely studied. Whether these genes and metabolites are significant in this process needs further verification.

## 5. Conclusions

*Hsp70* family genes (such as *Hsp68*, *Hsp70 A1*, and *Hsp70*), small peptides (such as tyrosine-isoleucine-histidine, phenylalanyltyrosine, tyrosine-phenylalanine-glutamic acid-lysine, and histidylleucine), γ-linolenic acid, and triglycerides may be involved in the process of silkworm eggs resisting high-temperature stress and induced oxidative stress, ensuring the smooth progress of life activities such as effective protein utilization and normal cell proliferation. In the future, we will use RNA interference (RNAi) or CRISPR-Cas9 technology to knock out *Hsp70* family genes or manipulate targeted metabolites to verify their direct impact on the high-temperature tolerance of silkworm eggs.

## Figures and Tables

**Table 1:** Screening results of DEGs.

Gene ID	log_2_Fold Change			KEGG Annotation
	CK vs. CG	CK vs. GW	CG vs. GW	
BMSK0007389	2.88	4.47	1.59	glycine N-methyltransferase
BMSK0014190	2.10	3.53	1.43	choline dehydrogenase
BMSK0006069	1.29	2.02	0.74	heat shock protein 68 isoform X1
BMSK0012507	4.05	5.52	1.49	heat shock protein 68
BMSK0015592	2.74	2.90	0.17	heat shock protein 19.9
BMSK0015594	2.77	3.37	0.62	heat shock protein 20.8
BMSK0015595	2.83	3.78	0.97	heat shock protein 20.4
BMSK0015668	2.59	3.60	1.03	heat shock protein 70 A1
BMSK0015669	3.70	5.67	1.99	heat shock protein 70
BMSK0015670	3.88	5.04	1.17	heat shock protein 70
BMSK0015671	3.88	5.44	1.58	heat shock protein 68
BMSK0015711	2.25	2.94	0.70	heat shock protein 23.7 precursor

CK, control group, and 28 °C; CG, conventional instant acid-impregnated, 46 °C, and 5 min; and GW, high-temperature instant acid-impregnated, 47.5 °C, and 7 min.

**Table 2:** Screening results of DMs.

Metabolite ID	log_2_Fold Change			Compound Annotations
	CK vs. CG	CK vs. GW	CG vs. GW	
MW0158501	0.85	2.09	1.24	Tyr-Ile-His
MW0110745	0.87	1.80	0.93	2-Hexadecanoylthio-1-ethylphosphorylcholine
MW0014926	0.74	1.65	0.91	N-(6-Aminohexanoyl)-6-aminohexanoate
MW0154890	0.25	1.08	0.83	Pantetheine
MW0109139	1.87	2.58	0.70	Phenylalanyltyrosine
MW0158567	0.65	1.20	0.55	Tyr-Phe-Glu-Lys
MEDP1869	0.47	0.97	0.50	Histidylleucine
MW0052451	−2.01	−4.43	−2.42	Docosahexaenoic Acid-d5
MEDP1388	−0.53	−2.65	−2.12	Carnitine C18:3-OH
MEDL02638	−0.39	−2.26	−1.87	Methylephedrine
MW0015407	−1.88	−3.72	−1.84	(9S,10S,12Z)-9,10-Dihydroxy-12-octadecenoic acid
MW0052740	−1.05	−2.50	−1.45	Ethyl tetradecanoate
MW0012192	−0.96	−2.38	−1.43	(9Z,12S,13R)-12,13-Dihydroxy-9-octadecenoic acid
MW0116986	−1.17	−2.24	−1.07	Cetylpyridinium
MW0006389	−1.31	−2.34	−1.03	Bisphenol A diglycidyl ether
MW0150223	−1.70	−2.31	−0.61	Glu-Val-Tyr-Asp
MW0144237	−0.80	−1.39	−0.60	Ac-DEVD-CHO
MW0144166	−0.86	−1.38	−0.52	9-keto Fluprostenol
MW0139561	−1.53	−2.05	−0.52	Resveratrol-3-O-sulfate
MW0052998	−3.56	−2.73	0.83	Gamma-Linolenic Acid
MW0069424	−0.99	−0.09	0.90	Triglyceride

CK, control group, and 28 °C; CG, conventional instant acid-impregnated, 46 °C, and 5 min; and GW, high-temperature instant acid-impregnated, 47.5 °C, and 7 min.

**Table 3:** Results of joint analysis of DEGs and DMs.

Gene (ID)	Compounds (ID)	Coefficient Rate	*p* Value
*Hsp68* (BMSK0012507)	Tyr-Ile-His (MW0158501)	0.84	5.58 × 10^−4^
	Phenylalanyltyrosine (MW0109139)	0.84	5.86 × 10^−4^
	Tyr-Phe-Glu-Lys (MW0158567)	0.96	5.36 × 10^−7^
	Histidylleucine (MEDP1869)	0.96	1.27 × 10^−6^
*Hsp70 A1* (BMSK0015668)	Tyr-Ile-His (MW0158501)	0.85	4.35 × 10^−4^
	Phenylalanyltyrosine (MW0109139)	0.89	1.25 × 10^−4^
	Tyr-Phe-Glu-Lys (MW0158567)	0.98	6.11 × 10^−9^
	Histidylleucine (MEDP1869)	0.96	7.14 × 10^−7^
*Hsp*70 (BMSK0015669)	Tyr-Ile-His (MW0158501)	0.84	6.10 × 10^−4^
	Tyr-Phe-Glu-Lys (MW0158567)	0.92	1.66 × 10^−5^
	Histidylleucine (MEDP1869)	0.92	2.42 × 10^−5^
*Hsp70* (BMSK0015670)	Tyr-Ile-His (MW0158501)	0.88	1.50 × 10^−4^
	Phenylalanyltyrosine (MW0109139)	0.90	8.01 × 10^−5^
	Tyr-Phe-Glu-Lys (MW0158567)	0.97	2.43 × 10^−7^
	Histidylleucine (MEDP1869)	0.92	2.94 × 10^−5^
*Hsp68* (BMSK0015671)	Tyr-Ile-His (MW0158501)	0.88	1.87 × 10^−4^
	Phenylalanyltyrosine (MW0109139)	0.84	6.58 × 10^−4^
	Tyr-Phe-Glu-Lys (MW0158567)	0.94	3.85 × 10^−6^
	Histidylleucine (MEDP1869)	0.90	6.00 × 10^−5^

## Data Availability

The original contributions presented in this study are included in the article/Supplementary Material. Further inquiries can be directed to the corresponding author.

## Supplementary Materials

The following supporting information can be downloaded at: https://www.mdpi.com/article/10.3390/insects16080862/s1, Table S1. Primer sequence. Table S2. Transcriptome sequencing data of silkworm eggs.
