# Supplementary material for: Transcriptomics and Metabolomics Combined to Analyze the Response Mechanism of Silkworm Eggs to High-Temperature Stress
Source: Insects. 2025 Aug 19;16(8):862. doi: 10.3390/insects16080862 (PMC12386467; doi:10.3390/insects16080862)
Supplement: Supplementary file 1 [file insects-16-00862-s001.zip › insects-3766548-supplementary.pdf]

Supplementary material

Table S1 Primer sequence.

Gene ID	Forward primer (5'-3')	Reverse primer (5'-3')
BMSK0007389	GTCGTACGTGGTGAACCTGT	TCCAGCATGCTCGTGAATGT
BMSK0014190	GAGCCGTCATCGCCAATAGA	GCATAGATTCGACGGAGGGG
BMSK0012507	CAGTCATCACCGTTCCTGCT	AAGCACATTCAGACCAGCGA
BMSK0015592	ACGAAATTGAGCGTCCTCGT	CCACGGGCGGTAGTATTCTC
BMSK0015594	TCTCGCCGGAAGAGATTTCG	CCCGTGCTCGTCTTTCTTCT
BMSK0015595	CGACGGGTACATCGTAGTGG	TGGTGAGTACCCCGTCTGAT
BMSK0015670	CAGGACGTCTTGTTGGTGGA	CTCTCCCTCGTACACCTGGA
BMSK0015711	AGGCTCCACAATCAAGACGG	ACCTTCCGCAGTCTTCACAG
GAPDH	TGTTGAGGGCTTGATGAC	ACCTTACCCACAGCTTTG

BMSK0007389, glycine N-methyltransferase; BMSK0014190, choline dehydrogenase; BMSK0012507, heat shock protein 68; BMSK0015592, heat shock protein 19.9; BMSK0015594, heat shock protein 20.8; BMSK0015595, heat shock protein 20.4; BMSK0015670, heat shock protein 70; and BMSK0015711, heat shock protein 23.7 precursor.

Table S2 –Transcriptome sequencing data of silkworm eggs.

Group	Total Reads	Reads mapped	Clean reads	Clean bases(G)	GC(%)	Q30(%)
	43158150	38491306(89.19%)	43158150	6.47	43.74	93.73
CK	43351234	38435878(88.66%)	43351234	6.50	43.18	93.51
	43936190	38586476(87.82%)	43936190	6.59	42.68	93.49
	45029040	39309427(87.30%)	45029040	6.75	42.10	93.35
CG	47043698	40946752(87.04%)	47043698	7.06	41.85	93.41
	44596878	39389759(88.32%)	44596878	6.69	43.03	93.42
	44775702	39365681(87.92%)	44775702	6.72	42.58	93.37
GW	46208018	41141366(89.04%)	46208018	6.93	43.70	93.80
	42932376	37874663(88.22%)	42932376	6.44	42.68	93.41

CK, control group, and 28 °C；CG, conventional instant acid-impregnated, 46 °C, and 5 min; and GW, high-temperature instant acid-impregnated, 47.5 °C, and 7 min.
